# Microbial neoformation of volatiles: implications for the estimation of post-mortem interval in decomposed human remains in an indoor setting

**DOI:** 10.1007/s00414-020-02436-4

**Published:** 2020-10-07

**Authors:** Ann-Sofie Ceciliason, M. Gunnar Andersson, Emma Lundin, Håkan Sandler

**Affiliations:** 1 Forensic Medicine, Department of Surgical Sciences; Uppsala University Hospital, Uppsala University, SE-751 85 Uppsala, Sweden; 2 Department of Forensic Medicine, The National Board of Forensic Medicine, Box 1024, SE-751 40 Uppsala, Sweden; 3 Department of Chemistry, Environment and Feed Hygiene, The National Veterinary Institute, SE-75189 Uppsala, Sweden

**Keywords:** Neoformation of ethanol, N-Propanol, 1-Butanol, Acetaldehyde, Post-mortem interval, Total body score

## Abstract

The objective of this study was to determine if a relationship between microbial neoformation of volatiles and the post-mortem interval (PMI) exists, and if the volatiles could be used as a tool to improve the precision of PMI estimation in decomposed human remains found in an indoor setting. Chromatograms from alcohol analysis (femoral vein blood) of 412 cases were retrospectively assessed for the presence of ethanol, N-propanol, 1-butanol, and acetaldehyde. The most common finding was acetaldehyde (83% of the cases), followed by ethanol (37%), N-propanol (21%), and 1-butanol (4%). A direct link between the volatiles and the PMI or the degree of decomposition was not observed. However, the decomposition had progressed faster in cases with microbial neoformation than in cases without signs of neoformation. Microbial neoformation may therefore act as an indicator of the decomposition rate within the early decomposition to bloating stages. This may be used in PMI estimation based on the total body score (TBS) and accumulated degree days (ADD) model, to potentially improve the model’s precision.

**Electronic supplementary material:**

The online version of this article (10.1007/s00414-020-02436-4) contains supplementary material, which is available to authorized users.

## Introduction

A deeper understanding of the decomposition process is important for several reasons, including estimating time of death or the post-mortem interval (PMI) and interpreting injuries, pathological changes, and toxicological results. The decomposition process is divided into two major chemical processes: autolysis (mediated by internal enzymes and chemicals) and putrefaction (mediated by bacteria). Putrefaction is fermentation in which microorganisms use organic compounds available in the dead body as electron acceptors to generate adenosine triphosphate for energy [1].

Ethanol is a well-known product of fermentation, although other volatiles are also produced post-mortem, such as acetaldehyde, acetone, 1-butanol, N-propanol, and isopropanol [2, 3]. The alcohols produced during decomposition depend on the microorganisms present and the substrates available [2]. Ethanol can also be used as a carbon or energy source by a variety of microorganisms [2, 4]. The origin of ethanol in a post-mortem blood sample may be difficult to interpret, especially in decomposed human remains and without knowledge of whether or not ante-mortem intake of ethanol occurred [5].

N-Propanol has been presented as a marker of putrefaction, as it reflects bacterial activity after death [6–9]. Another alcohol that is a possible marker of putrefaction is 1-butanol, often produced in parallel with N-propanol and ethanol [8–10]. In cases where ethanol is produced by common yeast (*Candida albicans*), N-propanol may not always be detected in post-mortem blood [11].

Acetaldehyde is the main metabolite of the oxidative pathway of ethanol and is eliminated rapidly in the living organism [12]. It has been detected in blood from decomposed human remains and may also be used as an indicator of putrefaction. However, the occurrence varies widely in different studies [13, 14], with some suggesting possible artefactual presence in blood samples [13]. Post-mortem-produced acetaldehyde and acetone have been detected in the absence of ethanol in germ-free mice [15].

In previous studies, ethanol, N-propanol, and 1-butanol were measurable after 1 day in experimental conditions [9, 10]. The neoformation of ethanol reached its maximum on day 10 post-mortem and remained stable to the end of the experiment at day 30 [10]. 1-Butanol was produced during the first 5 days. N-Propanol was produced increasingly, using amino acids as a substrate when the amount of carbohydrates declined [10]. An earlier in vitro study on post-mortem blood at room temperature indicated that the ethanol concentration increased rapidly until day 5, then stabilised to a maximum on day 15, and thereafter slowly decreased [16]. A general concordance with time was indicated in another study carried out under controlled experimental conditions, where an increase in ethanol neoformation was seen on day 2 and day 4 in post-mortem blood [17]. Despite a large number of published articles on ethanol and its microbial neoformation in human remains, reports on possible relation to the PMI or the degree of decomposition are still limited. N-Propanol and 1-butanol are mainly investigated as possible indicators of microbial neoformation of ethanol. The microbial communities within the decomposing body have an important role in PMI estimation [1]. Current research has shown promising results, estimating the PMI of decomposed bodies with an error as small as ± 2 days [1].

The objective of this study was to determine if microbial neoformation of volatiles (i.e., ethanol, N-propanol, 1-butanol, or acetaldehyde) was associated with the degree of decomposition or could occur without external signs. Another objective of this study was to determine if there was a relationship between microbial neoformation of volatiles and the PMI and if the volatiles could be used as a tool to increase the precision in PMI estimation of decomposed human remains found in an indoor setting.

## Materials and methods

### Forensic autopsy cases

The dataset in this study comprised the results of 412 forensic autopsy cases collected during 2011 to 2017 at the Departments of Forensic Medicine in Uppsala and Gothenburg, Sweden. All cases had femoral vein blood analysed for ethanol and were also assessed for external decomposition changes and scored according to the total body score (TBS) method [18, 19] during autopsy. Signs of external decomposition were present in approximately 49% of the cases. The inclusion criteria were that the deceased was an adult (> 18 years) found in an indoor setting with a known time of death. Submerged bodies (e.g., in a bathtub), burned bodies, and bodies with major trauma were not included due to possible influence on decomposition rate [20–22].

### Sampling, alcohol analysis, and classification of microbial neoformation

All blood samples had potassium fluoride (1–2% solution) added to stabilise the sample and inhibit degradation [5, 6]. All analyses were performed at the National Board of Forensic Medicine’s Department of Forensic Toxicology in Linköping, Sweden. A headspace gas chromatography with the flame ionisation detection (HS-GC-FID) method was used to analyse the femoral vein blood for alcohols and other volatiles (e.g., acetaldehyde, acetone) [23, 24]. This method is routinely used to quantify ethanol, acetone, isopropanol, and methanol, and is accredited by Swedac in accordance with an international standard (ISO/IEC 17025). The limit of detection is 0.1 mg/ml for all four substances. The chromatograms of the 412 cases were examined for the presence of ethanol, N-propanol, 1-butanol, and acetaldehyde in the femoral vein blood. The peak height for each detected substance was measured and normalised against the peak height of the internal standard (tert-butanol); the ratio was used as a proxy for the relative amount of the corresponding substance. The peak height of ethanol was also measured—even though the concentration was available—if the concentration was 0.1 mg/ml or higher.

Detected ethanol in the femoral vein blood was assumed to be partially or entirely neoformated (post-mortem-formed), provided that N-propanol and/or 1-butanol was detected in the same sample. If no ethanol was detected in a blood sample, but N-propanol and/or 1-butanol was, this was considered to represent microbial neoformation, i.e., the classification of microbial neoformation was based on the occurrence of N-propanol and/or 1-butanol.

Cases considered to have signs of microbial neoformation were further evaluated for possible ante-mortem intake of alcoholic beverages. The police reports were examined for testimonies of drinking and signs of ante-mortem intake (e.g., excessive amounts of alcoholic beverages, both empty and full canisters in the residence, canisters or glasses close to the body). Cases where acute alcohol intake was ruled as the cause of death or contributing to the death (as reported in the death certificates) were also classified as having ante-mortem intake of alcoholic beverages. In this retrospective study setup, further discrimination regarding confirmed ante-mortem intake of ethanol was not possible (additional toxicological analyses, for example, analysis of ethyl glucuronide).

### Ambient temperature, accumulated degree days, and morgue time

The indoor ambient temperature was approximately + 21 to + 22 °C, with a typical range of + 18 to + 23 °C due to building regulations [25]. The dead bodies were found in either a house or an apartment, resulting in limited access for insects. Cases where death occurred at a hospital were also included in the study and the ambient temperature in these cases was assumed to be similar (i.e., + 21 to + 22 °C). Shortly after death or when the dead body was discovered, the body was transported to a morgue facility (at the hospital or at the Department of Forensic Medicine) before autopsy. The temperature at the morgue was + 5 ± 1 °C. The majority of cases underwent autopsy after 2 to 6 days (median of 3 days) of storage at the morgue facility. The morgue time was defined as the time interval between the date of discovery of the dead body and the date of autopsy. Accumulated degree days (ADD) were calculated for each case and included the morgue time.

1 $$ \mathrm{ADD}=\mathrm{PMI}\times {T}_{\mathrm{site}}+\mathrm{MI}\times {T}_{\mathrm{morgue}} $$

MI is the morgue time, *T*_site_ is the temperature when and where the body was found, and *T*_morgue_ is the morgue temperature.

### The post-mortem interval (PMI)

The PMI was defined from the recorded time of death to the time the deceased was found. The recorded time of death was estimated based on witness statements of when last seen alive. There may be a systematic overestimation of the PMI, as the time of death does not necessarily follow directly after when a person was last seen alive. In cases where there was circumstantial information in police reports about the date of the most recent newspaper left in the mailbox, the latest registered phone call, receipts, or expiration dates on food or milk stored in the refrigerator, this information was also used to estimate a probable time of death.

### Assessing the degree of decomposition

The total body score (TBS) method is based on assessing the degree of decomposition in three different anatomical regions of the body (i.e., partial body score: head and neck, trunk and pelvis, limbs), scored independently based on the presence of external visual signs of decomposition. The three partial body scores are added together, resulting in a TBS reflecting the whole body’s degree of decomposition [18].

In this study, the degree of decomposition was scored at the autopsy, and the scores written down on a body chart, including both front and back view of the body. For each of the partial body scores, an average is calculated in cases with uneven decomposition (e.g., taking into account possible differences between the front and back of the body). The scale starts at 0 points (fresh or no decomposition changes) and ends at 32 points (fully skeletonised body) [19]. The same descriptions were applied for each point as in the original study by Megyesi et al. [18].

### Statistics

Microsoft Excel 2013 was used for database handling and descriptive statistical analysis. Linear regression analysis was performed in R 3.5.1 (https://www.R-project.org/) using the *lm* package.

## Results

The chromatograms from the alcohol analyses (femoral vein blood) of 412 forensic autopsy cases were retrospectively evaluated for ethanol, N-propanol, 1-butanol, and acetaldehyde. This section begins with an overall description of the cases and continues with data on the occurrence of alcohols and acetaldehyde. Further, we seek to determine if there was an association between neoformation of alcohols or acetaldehyde and the PMI and/or degree of decomposition. Lastly, we demonstrate a novel way of possibly modifying the TBS/ADD model for PMI estimation, based on the presence of microbial neoformation.

### Overall description

The median age at death in the complete dataset was 59 years (range 19 to 94 years) and the female to male ratio was 1:4.

The complete dataset was divided into two groups: non-decomposition cases (*n* = 209) and external decomposition cases (*n* = 203). The PMI ranged from 0 to 6 days (median 1 day) in the non-decomposition group, while the PMI ranged from 0 to 106 days (median 4 days) in the decomposition group, with only 5 cases of ≥ 30 days. Generally, short PMIs were more frequent than long PMIs.

TBS ranged from 0 to 18. A total of 209 cases lacked visual external signs of decomposition, i.e., TBS 0. Moreover, the majority of the cases (*n* = 176) with signs of external decomposition were within the early decomposition stage, corresponding to TBS 1–8 (greenish discolouration of skin, marbling, skin slippage). Another 12 cases were within the transition into bloating stage, approximately TBS 8 to 10, and a further 13 cases were within the bloating stage, approximately TBS 10 to 13. Only two cases had reached higher TBS: one case with TBS 14.5, within the active decomposition stage (post-bloat, brown to black discolouration of skin), and one case with TBS 18, in the transition into advanced decomposition stage (presence of partial desiccation).

Neoformation of ethanol was indicated in 85 of 203 cases (42%) exhibiting external signs of decomposition and, as comparison, in 6 of 209 cases (3%) without visible decomposition. In total, 91 (22%) of all 412 were classified as having microbial neoformation based on the findings of N-propanol and/or 1-butanol in femoral vein blood sample. After adjusting for suspected ante-mortem intake of alcoholic beverages, the neoformation group encompassed 56 cases.

Acetaldehyde was the most common volatile, found in 90 of 91 (99%) neoformation cases and 254 of 321 (79%) of the cases without neoformation. Ethanol was detected in 151 cases (37%), N-propanol in 86 cases (21%), and 1-butanol in 18 cases (4%) within the complete dataset. Femoral vein blood with no volatiles detected was seen in 64 cases (15.5%).

**Fig. 1:**
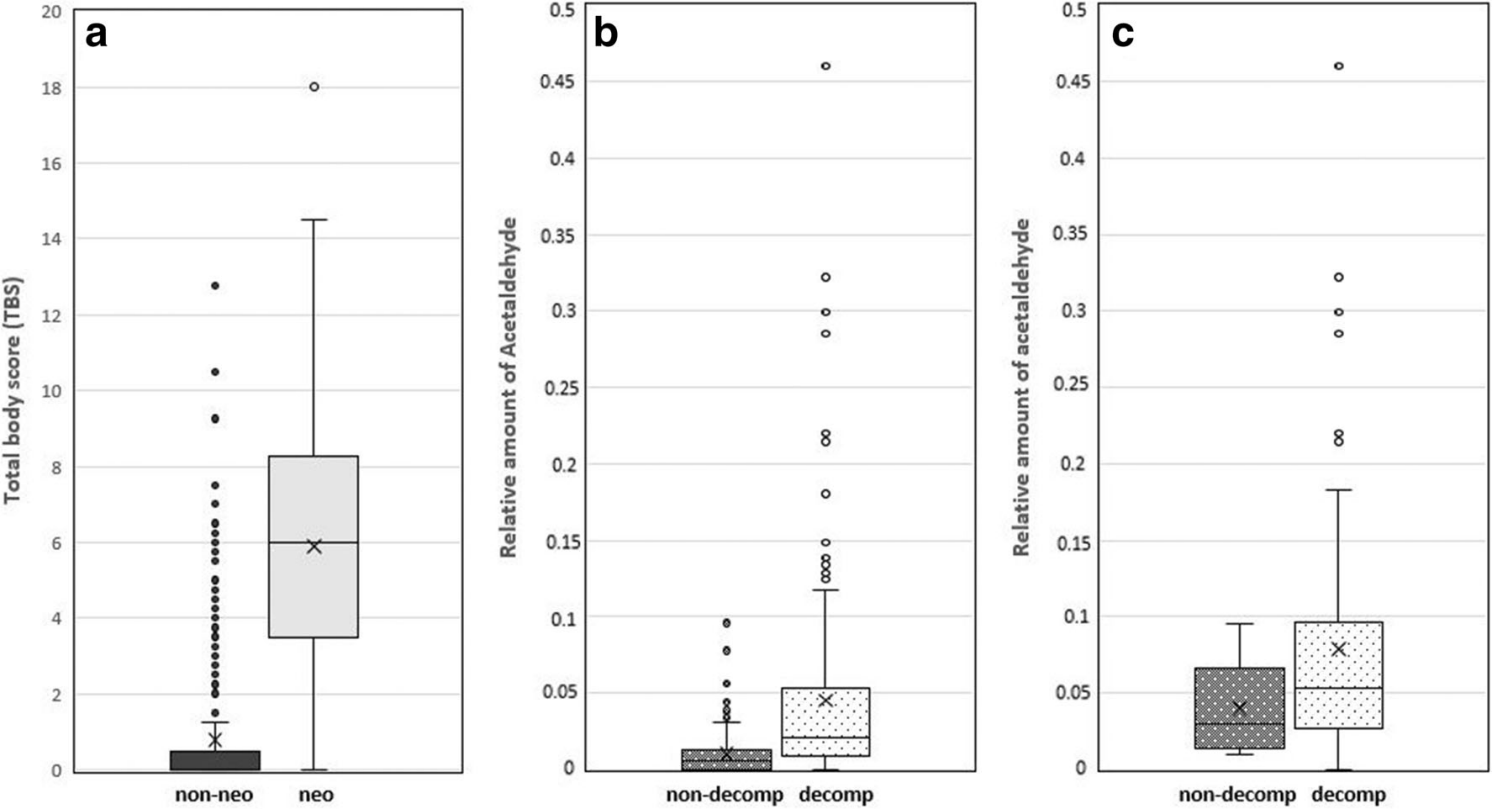
**a** The distribution of total body score (TBS) among cases without neoformation of volatiles (*n* = 321, non-neo) and cases with neoformation (*n* = 91, neo) based on detected N-propanol and/or 1-butanol in femoral vein blood. **b** The distribution of the relative amount of acetaldehyde in femoral vein blood among cases without external decomposition (*n* = 209, non-decomp) and cases with external decomposition (*n* = 203, decomp). **c** The distributions of the relative amount of acetaldehyde among cases with neoformation (*n* = 91) divided into non-decomposition cases (*n* = 6) and decomposition cases (*n* = 85). The horizontal lines represent medians. The crosses represent means. The top and bottom box lines show the first and third quartiles. The whiskers show the maximum and minimum values, with the exception of outliers (circles)

An apparent difference was seen between the cases without neoformation (*n* = 321) and the neoformation cases (*n* = 91) concerning the degree of decomposition, i.e., TBS, as visualised in Fig. 1a. TBS was generally higher in the neoformation cases, although several outliers were seen in the group of cases without neoformation.

A higher relative amount of acetaldehyde was seen within the decomposition group, in comparison with cases without decomposition (Fig. 1b). When looking only at the cases with neoformation (*n* = 91), a higher relative amount of acetaldehyde seemed to be associated with external decomposition (Fig. 1c).

### Presence of neoformation in relation to cause of death and/or known disease

In the non-decomposition group (*n* = 209), neoformation was indicated in femoral vein blood from 3 of 93 cases of natural cause of death (e.g., cardiovascular disease, cirrhosis, cancer), as well as in 3 of 81 cases of deaths from intoxication (i.e., overdose of opioids, antipsychotics, sedatives, antidepressants, or amphetamines). Neoformation was not indicated in femoral vein blood from the 29 cases of violent deaths, minor trauma (e.g., subdural hematomas, exsanguination due to knife wounds, asphyxia due to strangulation or hanging), or from the 6 cases of undetermined cause of death. Within the non-decomposition group, 21 cases had known diabetes mellitus (type 1 or 2) and 19 cases had a known infection (e.g., pneumonia, endocarditis, or myocarditis). None of those cases had signs of neoformation of ethanol in femoral vein blood.

In the decomposition group (*n* = 203), neoformation was indicated in the femoral vein blood from 54 of 134 cases of natural death, 17 of 42 cases of intoxications, 3 of 11 violent deaths, and 11 of 16 cases of undetermined cause of death. Within the decomposition group, 6 of 26 cases of known diabetes mellitus, as well as 12 of 25 cases with known infection, had signs of neoformation of ethanol in femoral vein blood.

### The association between the TBS or PMI and the detected volatiles

**Fig. 2:**
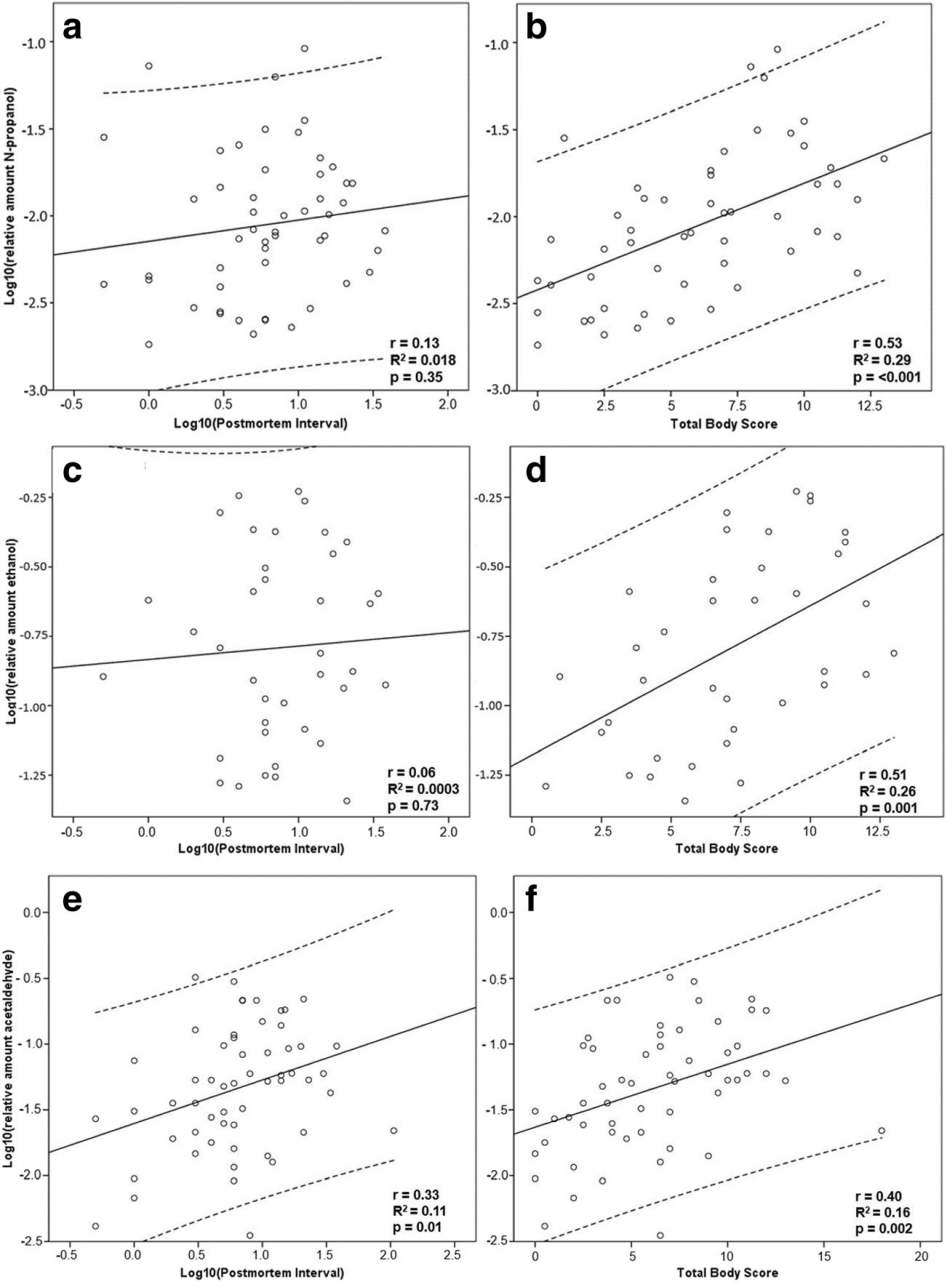
Scatter plots illustrating the correlation and linear regression in the neoformation group adjusted for ante-mortem intake (*n* = 56). A total of 52 cases were positive for N-propanol (**a**, **b**), 38 cases were positive for ethanol (**c**, **d**), and 56 cases were positive for acetaldehyde (**e**, **f**). Solid lines represent linear regression and dashed lines represent the 95% confidence interval

Scatter plots (Fig. 2) were created to visualise the possible association between the PMI or the degree of decomposition, in our study measured as TBS, and the relative amounts of N-propanol, ethanol, and acetaldehyde. The group of 56 neoformation cases was evaluated. Log_10_ transformation of the relative amounts of N-propanol, 1-butanol, ethanol, and acetaldehyde, as well as of the PMI, was applied to better visualise a possible linear relationship. When evaluating the association between TBS and the detected volatiles, the linear regression indicated a high significance level (with exception for 1-butanol), but low *R*^2^ values (0.16 to 0.29) and the Pearson correlation test indicated a positive weak to moderate linear relationship (*r* = 0.40–0.53). Furthermore, the results indicated no or weak linear relationship between the PMI and the detected volatiles (Pearson’s *r* = 0.06 to 0.33), with very low *R*^2^ values (0.0003 to 0.11, linear regression). The cases with 1-butanol were few in number (*n* = 13) and therefore harder to evaluate. Pearson’s *r* for log_10_PMI and TBS was 0.12 and 0.23, respectively. Linear regression resulted in log_10_PMI *R*^2^ = 0.01, *p* = 0.69, and TBS *R*^2^ = 0.05, *p* = 0.45 (figures not shown).

### The TBS/ADD model

The scatter plots (Fig. 2) did not suggest any apparent *linear* relationship between neoformation of ethanol, N-propanol, 1-butanol, or acetaldehyde and the PMI in the group of neoformation cases (*n* = 56, i.e., cases without suspected ante-mortem ethanol intake). Based on these findings, our hypothesis is that the relative amounts of volatiles found in a femoral vein blood sample may reflect the rate of decomposition. The volatiles were probably not accumulated during the early stages of decomposition, although production may temporarily be higher than elimination.

We further analysed the complete dataset (*n* = 412) with respect to the established TBS/ADD model, i.e., TBS as a function of ADD used in PMI estimation of decomposed human remains [18, 19]. We used a linear regression model in which the four volatiles were included as factor variables, i.e., as presence or absence in the blood sample. Our results indicated that the rate of decomposition was higher when microbial neoformation of volatiles was present in a dead body, yielding a higher TBS with similar ADD compared with that in cases without signs of neoformation.

With increasing TBS, the time period required to reach the next TBS score point also increases. It would not be enough that the decomposition process started at a higher rate in cases with neoformation; the rate must also have proceeded at a higher rate to be ahead of the cases without neoformation. We assume that microbial neoformation is an event early in the decomposition process and that there will not be a detectable difference in later decomposition rate between cases with or without neoformation.

When comparing the linear regression model TBS~log_10_ADD with the neoformation linear regression model TBS~log_10_ADD + acetaldehyde + neoformated ethanol + N-propanol + 1-butanol, we also observed that the precision of the model increased, indicated by a lower residual standard error (1.75 compared with 2.07). We repeated the two linear regression analyses with log_10_PMI instead of log_10_ADD. Similar results were achieved, but with a slightly higher residual standard error (results not shown). This may be explained by the fact that ADD, in our model, included the morgue time (i.e., the time the dead body is kept in a cold environment, the interval between time of discovery, and the time of autopsy). A high significance level was noted for 1-butanol and neoformated ethanol together with log_10_ADD or log_10_PMI.

The complete dataset of 412 cases includes several cases with a PMI < 1 day (*n* = 132). When excluding those cases and then comparing the two models, we saw rather similar results. Cases with a very short PMI (< 1 day) did not impact on the precision of the linear regression model (results not shown).

**Fig. 3:**
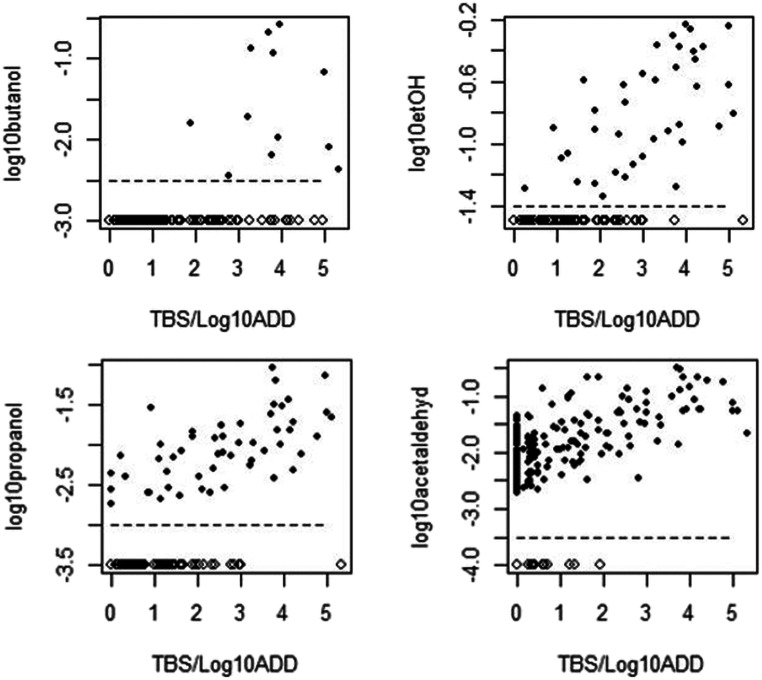
The rate of decomposition (calculated as TBS/log_10_ADD) illustrated for the four volatiles: 1-butanol (log_10_butanol), neoformated ethanol (log_10_etOH), N-propanol (log_10_propanol), and acetaldehyde (log_10_acetaldehyd). Solid black circles indicate cases with detected volatiles. Below the dashed lines, the open circles indicate cases without detected volatiles

Our hypothesis seemed to be supported by the results. Figure 3 illustrates the rate of decomposition when calculated as TBS/log_10_ADD for each volatile evaluated in this study. Cases with data points found in the right-hand part of the scatter plot (in Fig. 3) indicate high decomposition rate, i.e., a high TBS and low ADD. There seemed to be a tendency toward generally higher amounts of volatiles detected in the femoral vein blood when the rate of decomposition was higher.

### A potential way to improve PMI estimates of decomposed human remains

Presence of volatiles indicating the rate of decomposition could be used in the TBS/ADD model to achieve an improved precision of the PMI estimates. One way to do this would be to take the relative amounts of neoformated ethanol, N-propanol, 1-butanol, and acetaldehyde, and add them up, calculating a log_10_ sum of the relative amounts of volatiles (log_10_volatiles). Then, the coefficients from the linear regression model TBS~log_10_ADD + log_10_volatiles could be used to get an estimate of ADD in a single case.

**Fig. 4:**
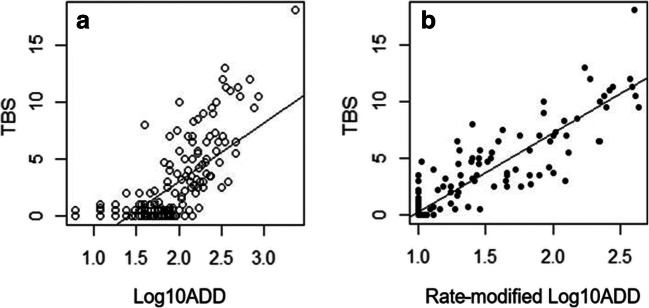
Comparison between the two regression models. **a** The original model: TBS~log_10_ADD, comprising the actual log_10_ADD values of each case. **b** The rate-modified model: TBS~rate-modified log_10_ADD model

We could think of log_10_ADD + log_10_volatiles as an ostensibly rate-modified log_10_ADD, representing not only ADD but also the amount of volatiles present in the blood sample. Figure 4 illustrates the original linear regression model TBS~log_10_ADD (Fig. 4a) in comparison with the rate-modified version, TBS~rate-modified log_10_ADD (Fig. 4b). As seen in Fig. 4b, the distribution of data points is narrowed, i.e., a better model fit is achieved.

## Discussion

After death, the unavoidable degradation of tissue and cell components begins and microorganisms from the gastrointestinal tract invade surrounding tissue and the vascular system. There is a microbial succession during the progress of decomposition that has been demonstrated to be predictable in nature and is possible to use as basis for PMI estimation [1]. Several different microorganisms (bacteria, yeasts, and moulds) that may be found in a dead body can synthetize ethanol as well as other volatiles (e.g., N-propanol and 1-butanol). It is known that the decomposition process [26, 27], substrate availability [11], and extensive trauma to the body [28] affect the extent of ethanol neoformation. However, ethanol is not always detected in decomposed human remains, probably due to elimination by microorganisms with the ability to use ethanol as a substrate [2, 4]. The specific combination of microorganism species present in a forensic case may also affect the magnitude of neoformation [2, 3, 6].

### Occurrence of neoformation of volatiles in human remains found in an indoor setting

In this study, we evaluated 412 forensic autopsy cases that were selected to include both decomposed bodies and bodies without external decomposition changes. The presence of N-propanol and/or 1-butanol was used as an indicator of microbial neoformation. In our dataset, 42% of the decomposed bodies had a femoral vein blood sample positive for N-propanol and/or 1-butanol, a considerable higher occurrence of neoformation than earlier reported (i.e., 18 to 22%) [26, 27]. However, a direct comparison with earlier studies is problematic due to the different ways to determine whether neoformation of alcohols has taken place. Both N-propanol and 1-butanol are considered to have a strong association with microbial neoformation of ethanol [9]. However, they do not signify a specific origin of ethanol, i.e., ante-mortem intake of ethanol is not excluded.

A recent study showed that microbial neoformation of ethanol may occur in a dead body before any external signs of decomposition are apparent [29]. In our study of cases without external signs of decomposition, positive findings of N-propanol and/or 1-butanol were found in 3% of the samples, indicative of microbial neoformation.

A possible caveat is that N-propanol and 1-butanol may come from ante-mortem intake of alcoholic beverages [30, 31]. Moreover, presence of fluoride ions in the blood inhibits the in vitro neoformation of ethanol [5, 6]. However, case reports indicate possible in vitro formation of ethanol in spite of addition of fluoride [32, 33].

Most cases classified as microbial neoformation in our study were positive for N-propanol in a femoral vein blood sample. The findings of 1-butanol were limited and the majority of those cases were also positive for N-propanol. An explanation for this low occurrence may be that a decomposing body in an indoor setting is not a favourable microbial environment for 1-butanol to be produced. The few cases in the dataset positive for 1-butanol generally seemed to have a numerically higher TBS. Therefore, it is also possible that 1-butanol is linked to specific stages of decomposition.

We observed a few cases with extensive decomposition and extended PMIs (up to 52 days) without N-propanol and/or 1-butanol. Some of these cases had detectable levels of ethanol in the femoral vein blood samples. It is known that N-propanol is not always produced during neoformation of ethanol [11]; hence, neoformation of ethanol cannot be ruled out even when N-propanol and 1-butanol are negative in a blood sample. Other volatiles, not evaluated in this study, may signify microbial neoformation.

### Relation of volatiles to the degree of decomposition and the post-mortem interval

The majority of the decomposed human remains in this study had a short PMI and were within the early stages of decomposition, i.e., they had low TBS. We observed that cases positive for N-propanol and/or 1-butanol generally had a higher TBS than those with negative femoral vein blood samples. One case with a TBS of 18 and a PMI of 106 days did not have any detectable ethanol in the femoral vein blood but was positive for 1-butanol. Ethanol may have been eliminated over time.

The correlation between TBS and ethanol or N-propanol was weak to moderate. In the linear regression model, the *R*^2^ was low, but a high significance level was indicated (Fig. 2). At the same time, the variation in ethanol or N-propanol amounts appeared not to be associated with the PMI. These results indicate a weak association between microbial neoformation and the PMI or TBS, respectively, in our model setting. However, this may be explained, to some extent, by our profile of cases.

### Occurrence of acetaldehyde

Acetaldehyde was present in 83% of all cases, usually coinciding with neoformation of ethanol, N-propanol, and/or 1-butanol. These cases also had significantly higher relative amounts of acetaldehyde than the cases without neoformation. The majority of cases negative for acetaldehyde also lacked signs of external decomposition. Acetaldehyde seemed to be somewhat associated with an increasing TBS, i.e., the degree of decomposition. However, a rather moderate correlation between TBS and the relative amount of acetaldehyde was noted. The occurrence of acetaldehyde is hard to assess fully. Still, acetaldehyde may be produced during the decomposition process due to bacterial activity and could also be produced by common yeast [34] as well as after oxidation of ethanol [35]. Acetaldehyde has also been suggested to be an artefact [13] and its occurrence varies widely between different studies [13, 14]. Acetaldehyde might be an interesting decomposition marker and perhaps also of importance in neoformation of ethanol when found in higher relative amounts.

### Effect of ambient temperature on microbial neoformation

Neoformation of ethanol increases at higher temperatures [17]. It has also been indicated that if a dead body is transported to a morgue within 4 h after death, neoformation of ethanol does not occur [36]. The ambient temperature is considered the factor with the largest impact on the decomposition rate and pattern [18, 37]. In this study, we had a narrow temperature span. The human remains were all found in indoor settings, meaning that the dead bodies had been confined to a more stable environment than an outdoor setting. Most of the bodies in this study were not refrigerated within 4 h after death. However, by taking the morgue time into account when calculating and using ADD, the possible effect of ambient temperature is incorporated into the model.

### Effect of cause of death or pre-existing disease on microbial neoformation

Bodies subjected to trauma with penetrating injuries may exhibit accelerated bacterial growth and, subsequently, neoformation of ethanol [38]. Dead bodies without external signs of decomposition, but with penetrating injuries, have been indicated to have neoformation of ethanol [29]. In our dataset, we did not include cases with extensive trauma. However, we included minor trauma such as knife wounds to the extremities or neck, blunt force trauma to the head, or asphyxia. In this group of violent deaths, no increase of cases with neoformation of ethanol was observed.

Diabetes may result in more abundant substrate (i.e., glucose) and induce a rise in ethanol levels [39]. This was not observed in our dataset, probably due to many diabetic patients having well-adjusted blood glucose levels. Furthermore, information on diabetes was retrieved only from police reports and many cases lacked information on known diseases.

Individuals with infection may be predisposed for microbial neoformation, as they are likely to have a larger burden of bacteria which may affect the rate of decomposition [37]. In the dataset, some cases with infections determined as cause of death or contributing to the death were evaluated; we found no increase of microbial neoformation in this group.

Natural causes of death (e.g., cardiovascular diseases, cirrhosis, cancer) and unnatural deaths due to intoxication (i.e., overdose of opioids, antipsychotics, sedatives, antidepressants or amphetamines) were rather similar within the dataset, with no distinction between cases with or without external decomposition changes. The type of intoxication may have interfered, but in our relatively small sample of cases, this was not further evaluated.

Undetermined causes of deaths within the decomposition group are mainly due to extensive decomposition changes making it difficult to determine a cause of death with certainty. The majority of those cases had signs of neoformation, presumably because of putrefaction. In the non-decomposition group, none of the undetermined cause of death cases had signs of neoformation.

Our results do not indicate a correlation between the cause of death or pre-existing disease (i.e., infections or diabetes) and occurrence of microbial neoformation.

### Factors affecting the level of neoformated ethanol

Microbial neoformation of ethanol is limited only by the substrate amount in a decomposing body (tissue and blood); the ethanol level may rise as far as is possible given the substrate (mainly glucose) available for fermentation by bacteria and yeast [40]. Glucose levels in the blood increase after death [2]. Previous research indicates that neoformation of ethanol takes place at the earliest 24 h after death [9, 10]. We may assume that during early decomposition, there is an abundance of substrate, creating a suitable environment for microbial neoformation inducing a higher rate of ethanol production [3]. Later on, the levels of ethanol peak and then decrease gradually over time. N-Propanol may be produced even during advanced stages of decomposition and could therefore increase after ethanol has peaked [3].

Several studies have investigated microbial neoformation of ethanol in vitro and commonly describe an initial increase in ethanol levels, then a stable level, followed by a decrease [10, 16, 17]. Translating this to the circumstances in our study might be difficult. Our dataset consists of forensic autopsy cases collected during a long period of time and with an expected natural biological variation between cases. In our model, signs of neoformation were observed in 4 cases with early external decomposition and a PMI of less than 1 day, as well as in 5 cases without external decomposition and a PMI of 1 day. The duration of the storage at the morgue varied between cases, which could affect neoformation of ethanol.

### Possibility of improving PMI estimation of decomposed human remains

We hypothesise that the microbial neoformation starts early (within the first days after death) and that the levels of ethanol and other volatiles may rise rapidly due to an abundance of substrate. A decrease in the levels of alcohols is seen later due to microbe-induced consumption and a decrease in available substrates. The presence of volatiles in a femoral vein blood sample may be used as a measure of the decomposition rate. All four volatiles assessed in this study may behave in a similar way, but have slightly different trajectories over time. It is likely that the dead body will have reached a specific stage of decomposition at a given point when a substrate is no longer available or elimination is faster than production. At this stage, microbial neoformation of alcohols would no longer be possible to use as an indicator of the decomposition rate. The relative amounts of acetaldehyde suggest a tendency toward higher values in cases with N-propanol and/or 1-butanol and increasing TBS, which may suggest an accumulation of acetaldehyde in later stages. Microbial neoformation of volatiles may be seen as an indicator of the microbial activity rather than as driving the decomposition process forward, at least up to a certain point in the decomposition of the dead body. The reason that we did not observe a direct link between the PMI and microbial neoformation of volatiles may be that there is not a linear relationship between the rate of decomposition and the PMI when looking at the entire range of PMIs in the dataset. Our indoor dataset, the range of PMIs, and the degrees of decomposition are typical for most forensic autopsies at our department. Thus, cases within the extended PMI range are very few, which could be considered a limitation.

The rate of decomposition seems to be higher in cases with microbial neoformation. This gives us the possibility to improve the TBS/ADD model, if we could adjust for differences in the rate of decomposition. The forensic application of the results in this study would be an ability to sort out cases where the decomposition rate may have been higher than expected, diminishing the risk of overestimation of the PMI. The studied volatiles are detected during routine forensic toxicological analysis and therefore readily accessible. Our results indicate that N-propanol and 1-butanol are the most promising indicators among the four volatiles evaluated, while ethanol levels may be affected to a varying extent by ante-mortem intake of alcoholic beverages. Acetaldehyde is the volatile most commonly detected in blood samples but is hard to interpret due to large variation in the observed levels, as well as difficulties in determining its origin in this model setup.

## Conclusions

This study yields novel information about the association between microbial neoformation of volatiles (i.e., ethanol, N-propanol, 1-butanol, and acetaldehyde) and the rate of decomposition. While the results suggest that any linear relationship between microbial neoformation and the PMI or degree of decomposition is weak, there is a potential way to improve PMI estimation of decomposed human bodies in our model setting. Microbial neoformation may act as an indicator of the *rate* of decomposition, enabling us to calculate a rate-modified log_10_ADD based on information about neoformated ethanol, N-propanol, 1-butanol, and acetaldehyde. When microbial neoformation is known, we may therefore increase the precision of the TBS/ADD model by including the volatiles. This model is probably most useful for cases exhibiting early decomposition changes or in the bloating stage.

The findings in this study may also be used in forensic toxicology, where the origin of ethanol is of importance. Here, it would enable prediction of whether or not detected ethanol is neoformated, as well as the amounts produced. For this, however, a prospective study based on a larger dataset would be required.

## Electronic supplementary material

ESM 1 (XLSX 53 kb)
